# Factors associated with negative pleural adenosine deaminase results in the diagnosis of childhood pleural tuberculosis

**DOI:** 10.1186/s12879-021-06209-1

**Published:** 2021-05-25

**Authors:** Xing-Fen Han, Chao Han, Feng Jin, Jun-Li Wang, Mao-Shui Wang

**Affiliations:** 1 Department of Tuberculosis, Shandong Provincial Chest Hospital, Cheeloo College of Medicine, Shandong University, Jinan, Shandong China; 2 Department of Geriatrics, Shandong Mental Health Center, Jinan, China; 3 Department of Thoracic Surgery, Shandong Provincial Chest Hospital, Cheeloo College of Medicine, Shandong University, Jinan, China; 4 Department of Lab Medicine, The Affiliated Hospital of Youjiang Medical University for Nationalities, Baise, China; 5 Department of Lab Medicine, Shandong Provincial Chest Hospital, Cheeloo College of Medicine, Shandong University, 46# Lishan Road, Jinan city, Shandong 250013 P. R. China

**Keywords:** Childhood pleural tuberculosis, Adenosine deaminase, Risk factor, Pleural effusion, Diagnosis

## Abstract

**Background:**

Until now, the influential factors associated with pleural adenosine deaminase (ADA) activity among children remain unclear. This retrospective study was therefore conducted aiming to investigate the factors associated with negative pleural ADA results in the diagnosis of childhood pleural tuberculosis (TB).

**Methods:**

Between January 2006 and December 2019, children patients with definite or possible pleural TB were recruited for potential analysis. Then, patients were stratified into two categories: negative pleural ADA results group (experimental group, ≤40 U/L) and positive pleural ADA results group (control group, > 40 U/L). Univariate and multivariate logistic regression analyses were performed to estimate risk factors for negative pleural ADA results.

**Results:**

A total of 84 patients with pleural TB were recruited and subsequently classified as experimental (*n* = 17) and control groups (*n* = 67). Multivariate analysis (Hosmer–Lemeshow goodness-of-fit test: χ^2^ = 1.881, df = 6, *P* = 0.930) revealed that variables, such as chest pain (age-adjusted OR = 0.0510, 95% CI: 0.004, 0.583), pleural total protein (≤45.3 g/L, age-adjusted OR = 27.7, 95% CI: 2.5, 307.7), pleural lactate dehydrogenase (LDH, ≤505 U/L, age-adjusted OR = 59.9, 95% CI: 4.2, 857.2) and blood urea nitrogen (≤3.2 mmol/L, age-adjusted OR = 32.0, 95% CI: 2.4, 426.9), were associated with negative pleural ADA results when diagnosing childhood pleural TB.

**Conclusion:**

Our findings demonstrated that chest pain, pleural total protein, pleural LDH, and blood urea nitrogen were associated with a negative pleural ADA result for the diagnosis of pleural TB among children. When interpreting pleural ADA levels in children with these characteristics, a careful clinical assessment is required for the pleural TB diagnosis.

**Supplementary Information:**

The online version contains supplementary material available at 10.1186/s12879-021-06209-1.

## Introduction

Childhood tuberculosis (TB) remains a serious health threat. In 2018, the World Health Organization (WHO) estimated that childhood TB comprised nearly 11% of all TB cases worldwide [1]. Moreover, childhood TB appears to be increasingly reported as a cause or comorbidity of acute pneumonia [2–4]. Pleural involvement is a common form of childhood TB. Although several reports have summarized the clinical characteristics of childhood pleural TB [5–8], the diagnosis of childhood pleural TB remains a challenge. For example, Cruz AT et al. found that pleural fluid cultures for TB were positive in 56% of enrolled childhood TB cases, and no case had acid-fast bacilli (AFB) smear-positive pleural fluid; in a previous study, we found 5.4% of children with pleural TB were AFB smear-positive, 14.3% were PCR positive, and 36.6% were culture-positive [7]. Although thoracoscopy has been proven to be a sensitive and safe tool for the detection of childhood pleural TB, it has limited usefulness due to a invasive procedure [9]. In a word, the diagnostic performance of routine TB assays remains unsatisfied. In addition, some novel TB assays, such as Xpert, also have relatively limited clinical use in the diagnosis of childhood pleural TB [10, 11].

Until now, several meta-analyses have investigated the diagnostic role of pleural adenosine deaminase (ADA) in the diagnosis of pleural TB in adults, with an approximately sensitivity and specificity of 92–93% and 90–92%, respectively [12–15]. Likewise, an increased level of pleural ADA is also considered as a diagnostic criteria of childhood pleural TB. Unfortunately, several factors were reported to have an influential effect on the level of pleural ADA, such as IgG4-related pleuritis, lymphoma, age, empyema and mycobacterial load [16–20]. However, until now, the influential factors associated with pleural ADA activity in childhood pleural TB remain unclear. To improve the usefulness of pleural ADA in childhood TB, this retrospective study was therefore conducted aiming to investigate the factors associated with negative pleural ADA results for the diagnosis of childhood pleural TB.

## Patients and methods

This study was conducted in accordance with the Helsinki Declaration and approved by the Ethics Committee of Shandong Provincial Chest Hospital. Due to the retrospective nature of this investigation and the anonymous nature of the data collection, written informed consent was waived by the Ethics Committee of Shandong Provincial Chest Hospital.

Between January 2006 and December 2019, children patients (≤ 15 years old) with suspected of pleural TB were recruited for potential analysis. Definite pleural TB was defined as the isolation of TB strains from mycobacterial cultures (sputum, pleural effusion, or pleural tissue), or the presence of pathological evidence (such as caseous necrosis, or Langhans’ giant cells). Possible pleural TB was diagnosed based on compatible clinical symptoms plus a positive result of TB assays (such as TB RT-PCR, acid-fast bacilli (AFB) smear, or both).

Pleural effusion were collected for analysis before starting anti-TB treatment or within 7 days of starting anti-TB treatment. The pleural ADA activity was measured colorimetrically using an ADA assay kit (Maccura, Chengdou, China) on a chemistry analyzer. The threshold of pleural ADA for childhood pleural TB was selected based on general expert opinions and most studies [13, 21, 22] and patients were then stratified into two categories: negative pleural ADA results group (referred as the experimental group, ≤40 U/L) and positive pleural ADA results group (referred as the control group, > 40 U/L). The demographic, clinical, laboratory, and radiographic features were collected from the electric medical records retrospectively.

SPSS version 16.0 (SPSS, Chicago, IL, USA) was used to perform the statistical analysis. All data were described as mean ± standard deviation (SD). Differences between the two groups were compared using Mann-Whitney *U* test or *t* test for continuous and χ^2^ test or Fisher exact test for categorical variables. The associations between the parameters were analyzed using the Spearman correlation test. Univariate logistic regression analysis was performed to estimate risk factors for negative pleural ADA results, and variables with *P* value < 0.1 were included for multivariate logistic regression analysis. Prior to multivariate regression analysis, continuous variables were transformed into categorical variables by receiver operating characteristic curve (ROC) analysis. Then, multivariate logistic regression analysis was performed and the corresponding odds ratios (OR) and 95% confidence interval (CI), adjusted by age were calculated [23]. In addition, the accuracy of the multivariate model was tested using the Hosmer-Lemeshow goodness-of-fit test. A *P* value < 0.05 was considered significant for the difference assessed.

## Results

### Characteristics of enrolled patients

During the study period, 1577 childhood TB patients were diagnosed in the center. Of them, 458 patients (29.0%) were diagnosed as pleural TB and 154 patients were confirmed as definite or possible pleural TB. Among the 154 patients with definite or possible pleural TB, 84 patients underwent pleural ADA assay, including definite (*n* = 69) or possible (*n* = 15) cases. Subsequently, these cases were classified as experimental (*n* = 17) and control groups (*n* = 67). Pathological evidence for TB were found in 27 cases (experimental, *n* = 5; control, *n* = 21) and microbiological diagnostic tests were as follows: mycobacterial culture (experimental, *n* = 14; control, *n* = 51), AFB (experimental, *n* = 1; control, *n* = 4), and TB RT-PCR (experimental, *n* = 8; control, *n* = 22). The experimental group consisted of 14 definite patients and 3 possible patients, and the control group consisted of 55 definite patients and 12 possible patients. The demographic data and clinical characteristics of children with pleural TB were presented in Table 1 and Supplementary Table 1. The mean age was 12.0 ± 3.3 years. Boys accounted for 66.7% (56 patients) and 53 (100%) were HIV-negative. The mean weight was 44.7 ± 15.7 Kg. Among the 84 children patients, 40 (47.6%) were from rural areas. The vital signs were as follows: blood pressure, 110.3 ± 12.6/68.5 ± 8.8 mmHg; heart rate, 98.4 ± 14.4 beats/min; respiratory rate, 22.5 ± 2.7 breaths/min; and temperature, 37.4 ± 1.0 °C.

**Table 1:** Univariate analysis of the demographic and clinical data associated with negative pleural ADA results in childhood pleural TB

	Total (n)	Pleural ADA (≤40 U/L)	Pleural ADA (>40 U/L)	*P* value	OR (95% CI)
N	84	17 (20.2%)	67 (79.8%)		
Pleural ADA (U/L)	60.3 ± 28.8	28.3 ± 9.1	68.0 ± 26.3		
Demographic characteristics					
Age (years)	12.0 ± 3.3	12.1 ± 2.7	12.0 ± 3.4	0.895	
Sex (male)	56 (66.7%)	11 (64.7%)	45 (67.2%)	0.848	
Weight (Kg)	44.7 ± 15.7	42.3 ± 13.0	45.3 ± 16.3	0.509	
Rural area	40 (47.6%)	10 (58.8%)	39 (58.2%)	0.114	
Symptoms					
Cough	47 (56.0%)	11 (64.7%)	36 (53.7%)	0.418	
Fever	76 (90.5%)	13 (76.5%)	63 (94.0%)	0.040	4.846 (1.072, 21.916)
Chest pain	43 (51.2%)	13 (76.5%)	30 (44.8%)	0.026	0.249 (0.074, 0.845)
Dyspnea	24 (28.6%)	3 (17.6%)	21 (31.3%)	0.272	
Sputum production	14 (16.7%)	2 (11.8%)	12 (17.9%)	0.547	
Cavity	1 (1.2%)	0 (0.0%)	1 (1.5%)	1.000	
Loculated effusion	14 (16.7%)	4 (23.5%)	10 (14.9%)	0.399	
Empyema	10 (11.9%)	4 (23.5%)	6 (9.0%)	0.110	
Effusion sites					
Left	33 (39.3%)	10 (58.8%)	23 (34.3%)	0.071	0.366 (0.123, 1.088)
Right	43 (51.2%)	6 (35.3%)	37 (55.2%)	0.148	
Both	8 (9.5%)	1 (5.9%)	7 (10.4%)	0.572	
Clinical Chemistry (pleural effusion)					
Total protein	48.5 ± 7.2	42.8 ± 9.0	49.9 ± 5.8	0.001	1.163 (1.061, 1.274)
Total bilirubin (mmol/L)	8.6 ± 5.6	11.3 ± 8.3	7.9 ± 4.4	0.050	0.914 (0.835, 1.000)
Glucose (mmol/L)	3.3 ± 1.5	3.9 ± 1.3	3.2 ± 1.6	0.105	
Lactate dehydrogenase (U/L)	876.7 ± 642.8	480.1 ± 271.3	968.7 ± 670.3	0.002	1.004 (1.002, 1.007)
Amylase (U/L)	29.6 ± 10.4	26.6 ± 10.9	30.4 ± 10.2	0.187	
Clinical Chemistry (serum)					
Total protein (g/L)	69.4 ± 7.1	68.6 ± 7.8	69.5 ± 6.9	0.590	
Albumin (g/L)	38.9 ± 4.4	38.8 ± 5.1	38.8 ± 4.4	0.936	
Blood urea nitrogen (mmol/L)	3.9 ± 1.2	3.1 ± 0.8	4.1 ± 1.2	0.005	2.367 (1.301, 4.307)
Creatinine (μmmol/L)	51.2 ± 15.1	51.0 ± 13.2	51.5 ± 15.8	0.954	
Glucose (mmol/L)	4.8 ± 0.6	4.7 ± 0.5	4.9 ± 0.7	0.412	
Lactate dehydrogenase (U/L)	216.5 ± 56.1	210.5 ± 55.3	218.2 ± 56.7	0.647	

*ADA* adenosine deaminase, *TB* tuberculosis, *OR* odds ratio, *CI* confidence interval

Among the enrolled children patients, 10 (11.9%) had a TB contact history and 11 (13.1%) were treated with surgical procedures. The mean transferred times between hospitals were 2.1 ± 0.9 and mean times of hospitalization were 1.8 ± 1.3. Most of them (49, 58.3%) were transferred from a teaching hospital. The symptoms complicated were as follows: fever (76, 90.5%), cough (47, 56.0%), chest pain (43, 51.2%), dyspnea (24, 28.6%), and sputum production (14, 16.7%). Radiographic findings showed that 1 patient (1.2%) had a cavity, 14 (16.7%) had loculated effusion, 33 (39.3%) had effusion on the left-side, 43 (51.2%) on the right-side, and 8 (9.5%) on the both-side. Sixty-two (73.8%) patients underwent thoracentesis before starting anti-TB treatment and 22 (26.2%) patients underwent it within seven days of starting anti-TB treatment. Out of the total of 84 study patients, 48 (57.1%) had pulmonary TB, 10 (11.9%) had empyema, 6 (7.1%) had tuberculous lymphadenitis, 2 (2.4%) had miliary TB 1 (1.2%) had bronchial TB, and 1 (1.2%) had tuberculous meningitis.

Other characteristics, such as clinical chemistry analysis (serum or pleural effusion), blood cell analysis, and flow cytometry analysis, were also showed in Table 1.

### Comparisons between experimental and control groups

For comparison of continuous variables between the two groups (experimental vs control group), Mann-Whitney *U* tests were used and the statistical analysis showed that differences in pleural total protein (*P* < 0.01), pleural lactate dehydrogenase (LDH, *P* < 0.01), and blood urea nitrogen (*P* < 0.01) were significant when comparing the two groups. The differences in other continuous variables did not reach significance (all *P* > 0.05). For dichotomous variables, chi-square analysis was performed, and the analysis suggested that difference between the two groups was significant in fever and chest pain (all *P* < 0.05). The differences in other continuous variables did not reach significance (all *P* > 0.05).

### Univariate and multivariate analysis

Univariate analysis was performed to estimate each risk factor for the negative pleural ADA results when diagnosing childhood pleural TB. It was found that pleural total protein (OR = 1.163, 95% CI: 1.061, 1.274), pleural LDH (OR = 1.004, 95% CI: 1.002, 1.007), and blood urea nitrogen (OR = 2.367, 95% CI: 1.301, 4.307), fever (OR = 4.846, 95% CI: 1.072, 21.916), and chest pain (OR = 0.249, 95% CI: 0.074, 0.845) were associated with the negative pleural ADA result in the diagnosis of childhood pleural TB (all *P* < 0.05).

To make the results as readily understandable as possible, continuous variables were converted into dichotomous categorical variables based on the cutoff points determined using ROC analysis, and the corresponding optimal cutoff values were 45.3 g/L, 505 U/L, and 3.2 mmol/L for pleural total protein, pleural LDH, and blood urea nitrogen, respectively. Further multivariate analysis (Hosmer–Lemeshow goodness-of-fit test: χ^2^ = 1.881, df = 6, *P* = 0.930) revealed that variables, such as chest pain (age-adjusted OR = 0.0510, 95% CI: 0.004, 0.583), pleural total protein (≤45.3 g/L, age-adjusted OR = 27.7, 95% CI: 2.5, 307.7), pleural LDH (≤505 U/L, age-adjusted OR = 59.9, 95% CI: 4.2, 857.2), and blood urea nitrogen (≤3.2 mmol/L, age-adjusted OR = 32.0, 95% CI: 2.4, 426.9), were associated with negative pleural ADA results in diagnosis of childhood pleural TB (Table 2).

**Table 2:** Age-adjusted OR for risk factors associated with negative pleural ADA results in childhood pleural TB

	Age-adjusted OR (95% CI)	*P* value
Chest pain	0.0510 (0.004, 0.583)	0.017
Pleural total protein (≤45.3 g/L)	27.7 (2.5, 307.7)	0.007
Pleural LDH (≤505 U/L)	59.9 (4.2, 857.2)	0.003
Blood urea nitrogen (≤3.2 mmol/L)	32.0 (2.4, 426.9)	0.009

*OR* odds ratio, *TB* tuberculosis, *ADA* adenosine deaminase, *CI* confidence interval, *LDH* lactate dehydrogenase

## Discussion

Pleural effusion is a common complication of pneumonia in children. Pleural TB is usually considered if the pleural ADA have a value of > 40 U/L [24]. However, it remains a significant proportion of children with pleural TB have a pleural ADA value under the threshold of 40 U/L [6]. In this study, several risk factors, such as absence of chest pain and higher values of pleural total protein, pleural LDH, and blood urea nitrogen were associated with negative pleural ADA results in children with pleural TB. To our best knowledge, this study is the first research investigating the association between variables and negative pleural ADA results, and we believe that our findings would aid to improve the diagnosis of pleural TB in children.

ADA is known as an enzyme which catalyses the conversion of adenosine to inosine and joins in the differentiation of lymphoid cells. Currently, several studies have investigated the factors influencing the pleural level of ADA. First, a high ADA activity is associated with a stimulated cellular immunity. Production of pleural ADA reflects an activation of T cells and monocytes in effusion [25]. In a previous study, it was demonstrated a positive correlation between pleural ADA level and CD4 lymphocyte counts [26]. Second, Kim SB et al. found that older age was significantly associated with low pleural ADA activity among patients with pleural TB [17]. Similarly, the association between age and pleural ADA was also reported in other studies [27–29]. A possible explanation for this is that aging declines human immunity, such as T cell function, macrophage number and functions [30–33]. As the above mentioned, age-related changes in the activity of ADA may be expected. Third, a typical tuberculous pleural effusion manifestation involves lymphocyte predominance. However, up to 10% of tuberculous effusions are neutrophil-dominant pattern, which means a lower level of pleural ADA activity [34]. Fourth, pleural ADA activity may be associated with a status of anti-TB treatment. Soedarsono S et al. found that the serum ADA level at the beginning of TB treatment was higher than the level at the end of intensive phase treatment, and then the study concluded that the serum ADA test can be used to evaluate the pulmonary TB treatment response [35]. In addition, an increased level of pleural ADA levels are also found in other etiologies, such as rheumatoid pleural effusion, bacterial pleural infection, mesothelioma, lung cancer, leukaemia, empyema, and lymphoma [20, 36]. Although these factors influencing the level of pleural ADA have been identified previously, our findings are inconsistent with these findings. This may be attributed to two reasons: a small sample size and a younger population recruited.

First, our findings found that the absence of chest pain is considered a risk factor of negative pleural ADA results. One possible explanation is that in a previous study, chest pain was more common in children with complicated community-acquired pneumonia and associated with post-operative death [37, 38]; in addition, several pleural effusion markers are associated with complicated effusion and serious outcomes (such as death), e.g. low pleural pH and glucose, and high pleural LDH activity [39]; therefore, it is thought that the chest pain is associated with a higher level of pleural ADA among children with pleural TB. Moreover, in terms of TB disease, chest pain was associated with the occurrence of pleural TB [40].

Second, a decreased level of blood urea nitrogen was associated with a negative pleural ADA result. This maybe inconsistent with the previous findings in adulthood [41]. Because, In adult patients with renal failure, haemodialysis is a confounding factor which could reduce the levels of ADA [42]. In fact, the exact mechanism of the association between blood urea nitrogen and pleural ADA in children patients remains unclear. This may be explained by a positive correlation between age and blood urea nitrogen [43]. However, to illustrate the point, further investigation is required.

Third, pleural protein and LDH indicated a degree of pleural inflammation, it is thought that a greater pleural inflammation would lead to more activated lymphocytes and ADA production. Therefore, a positive association is built between pleural ADA and other inflammatory biomarkers. For example, a previous study suggested a significant correlation between pleural ADA and pleural protein and LDH [29]. In addition, Bielsa S et al. found that pleural ADA < 35 U/L was associated with pleural LDH levels < 500 U/L [44]. These finding are similar to our observation of the study.

Our study also had some limitations. First, the ADA criteria (40 U/L) may be high for the diagnosis of pleural TB in areas with high TB prevalence. Second, this was a single centre study, it thus may only reflect a local epidemiological situation. Third, the study had a retrospective nature and some clinical data (e.g., pleural cell subsets, isoenzyme activity (ADA1 and ADA2)) could not be obtained. Therefore, further studies are needed to determine the mechanisms involving low ADA activity among patients with pleural TB, and future large prospective studies would be needed to validate the above findings.

## Conclusions

The study found that several variables, such as chest pain, pleural total protein, pleural LDH, and blood urea nitrogen, have been identified as risk factors associated with negative pleural ADA results in the diagnosis of pleural TB among children. A false pleural ADA result may lower the suspicion of pleural TB and result in delayed diagnosis and anti-TB treatment. Therefore, when interpreting pleural ADA levels in children with such characteristics for the diagnosis of pleural TB, a careful clinical assessment is required.

## Supplementary Information

**Additional file 1.**

## Data Availability

The data analyzed in this study can be accessed by sending a request to the corresponding author.

## Abbreviations

ADA: Adenosine deaminase
TB: Tuberculosis
WHO: World Health Organization
SD: Standard deviation
ROC: Receiver operating characteristic curve
OR: Odds ratios
CI: Confidence interval
LDH: Lactate dehydrogenase

## References

[1] WHO (2019). Global tuberculosis report 2019.

[2] Oliwa JN, Karumbi JM, Marais BJ, Madhi SA, Graham SM (2015). Tuberculosis as a cause or comorbidity of childhood pneumonia in tuberculosis-endemic areas: a systematic review. Lancet Respir Med.

[3] Martinez L, le Roux DM, Barnett W, Stadler A, Nicol MP, Zar HJ (2018). Tuberculin skin test conversion and primary progressive tuberculosis disease in the first 5 years of life: a birth cohort study from Cape Town, South Africa. Lancet Child Adolesc Health.

[4] Marangu D, Zar HJ (2019). Childhood pneumonia in low-and-middle-income countries: an update. Paediatr Respir Rev.

[5] Utine GE, Ozcelik U, Kiper N, Dogru D, Yalcn E, Cobanoglu N, Pekcan S, Kara A, Cengiz AB, Ceyhan M (2009). Pediatric pleural effusions: etiological evaluation in 492 patients over 29 years. Turk J Pediatr.

[6] Cruz AT, Ong LT, Starke JR (2009). Childhood pleural tuberculosis: a review of 45 cases. Pediatr Infect Dis J.

[7] Wang JL, Zhao GW, Zhang ZQ, Wang XF, Wang MS (2015). Clinicopathologic characteristics of pediatric tuberculous pleural effusion: a retrospective analysis of 112 consecutive cases. Eur Rev Med Pharmacol Sci.

[8] Bayhan GI, Sayir F, Tanir G, Tuncer O (2018). Pediatric pleural tuberculosis. Int J Mycobacteriol.

[9] Wang M, Han C, He Y (2019). Diagnostic role of medical thoracoscopy in childhood pleural tuberculosis. Sci Rep.

[10] Kohli M, Schiller I, Dendukuri N, Dheda K, Denkinger CM, Schumacher SG, Steingart KR (2018). Xpert((R)) MTB/RIF assay for extrapulmonary tuberculosis and rifampicin resistance. Cochrane Database Syst Rev.

[11] Huo ZY, Peng L (2018). Is Xpert MTB/RIF appropriate for diagnosing tuberculous pleurisy with pleural fluid samples? A systematic review. BMC Infect Dis.

[12] Palma RM, Bielsa S, Esquerda A, Martinez-Alonso M, Porcel JM (2019). Diagnostic accuracy of pleural fluid adenosine deaminase for diagnosing tuberculosis. Meta-analysis of Spanish Studies. Arch Bronconeumol.

[13] Liang QL, Shi HZ, Wang K, Qin SM, Qin XJ (2008). Diagnostic accuracy of adenosine deaminase in tuberculous pleurisy: a meta-analysis. Respir Med.

[14] Goto M, Noguchi Y, Koyama H, Hira K, Shimbo T, Fukui T (2003). Diagnostic value of adenosine deaminase in tuberculous pleural effusion: a meta-analysis. Ann Clin Biochem.

[15] Aggarwal AN, Agarwal R, Sehgal IS, Dhooria S (2019). Adenosine deaminase for diagnosis of tuberculous pleural effusion: a systematic review and meta-analysis. PLoS One.

[16] Tamura K, Suzuki M, Ishii S, Takasaki J, Naka G, Iikura M, Izumi S, Takeda Y, Hojo M, Sugiyama H (2020). IgG4-related disease with elevated adenosine deaminase in pleural effusion diagnosed clinically using thoracoscopy under local anesthesia and FDG-PET-CT. Respir Med Case Rep.

[17] Kim SB, Shin B, Lee JH, Lee SJ, Lee MK, Lee WY, Yong SJ, Kim SH (2020). Pleural fluid ADA activity in tuberculous pleurisy can be low in elderly, critically ill patients with multi-organ failure. BMC Pulm Med.

[18] Nagayasu A, Kubo S, Nakano K, Nakayamada S, Iwata S, Miyagawa I, Fukuyo S, Saito K, Tanaka Y (2018). IgG4-related Pleuritis with elevated adenosine deaminase in pleural effusion. Intern Med.

[19] Kim CH, Lee J, Cliff JM, Toulza F, Smith S, Yoo SS, et al. Mycobacterial load affects adenosine deaminase 2 levels of tuberculous pleural effusion. J Inf Secur. 2015. 10.1016/j.jinf.2015.05.015 26049138

[20] Porcel JM, Esquerda A, Bielsa S (2010). Diagnostic performance of adenosine deaminase activity in pleural fluid: a single-center experience with over 2100 consecutive patients. Eur J Intern Med.

[21] Light RW (2010). Update on tuberculous pleural effusion. Respirology..

[22] Porcel JM (2009). Tuberculous pleural effusion. Lung..

[23] Zhang W, Han C, Wang MS, He Y (2018). Characteristics and factors associated with treatment delay in pleural tuberculosis. QJM..

[24] Fischer GB (2016). Pleural effusions in children from southern Brazil. Paediatr Respir Rev.

[25] Ungerer JP, Oosthuizen HM, Retief JH, Bissbort SH (1994). Significance of adenosine deaminase activity and its isoenzymes in tuberculous effusions. Chest..

[26] Baganha MF, Pego A, Lima MA, Gaspar EV, Cordeiro AR (1990). Serum and pleural adenosine deaminase. Correlation with lymphocytic populations. Chest..

[27] Korczynski P, Klimiuk J, Safianowska A, Krenke R (2019). Impact of age on the diagnostic yield of four different biomarkers of tuberculous pleural effusion. Tuberculosis (Edinb).

[28] Lee SJ, Kim HS, Lee SH, Lee TW, Lee HR, Cho YJ, Jeong YY, Kim HC, Lee JD, Hwang YS (2014). Factors influencing pleural adenosine deaminase level in patients with tuberculous pleurisy. Am J Med Sci.

[29] Tay TR, Tee A (2013). Factors affecting pleural fluid adenosine deaminase level and the implication on the diagnosis of tuberculous pleural effusion: a retrospective cohort study. BMC Infect Dis.

[30] Pawelec G, Barnett Y, Forsey R, Frasca D, Globerson A, McLeod J, Caruso C, Franceschi C, Fulop T, Gupta S (2002). T cells and aging, January 2002 update. Front Biosci.

[31] Bektas A, Schurman SH, Sen R, Ferrucci L (2017). Human T cell immunosenescence and inflammation in aging. J Leukoc Biol.

[32] Linehan E, Fitzgerald DC (2015). Ageing and the immune system: focus on macrophages. Eur J Microbiol Immunol (Bp).

[33] Ogawa T, Kitagawa M, Hirokawa K (2000). Age-related changes of human bone marrow: a histometric estimation of proliferative cells, apoptotic cells, T cells, B cells and macrophages. Mech Ageing Dev.

[34] Rahman NM, Chapman SJ, Davies RJ (2004). Pleural effusion: a structured approach to care. Br Med Bull.

[35] Soedarsono S, Prinasetyo K, Tanzilia M, Nugraha J (2020). Changes of serum adenosine deaminase level in new cases of pulmonary tuberculosis before and after intensive phase treatment. Lung India.

[36] Hsu WH, Chiang CD, Huang PL (1993). Diagnostic value of pleural adenosine deaminase in tuberculous effusions of immunocompromised hosts. J Formos Med Assoc.

[37] Byington CL, Spencer LY, Johnson TA, Pavia AT, Allen D, Mason EO, Kaplan S, Carroll KC, Daly JA, Christenson JC (2002). An epidemiological investigation of a sustained high rate of pediatric parapneumonic empyema: risk factors and microbiological associations. Clin Infect Dis.

[38] Tsai YM, Lin YL, Chang H, Lee SC, Huang TW (2019). Clinical outcome and risk factors for emergency department adult patients with thoracic empyema after video-assisted thoracic surgical procedure. Surg Infect.

[39] Segura RM (2004). Useful clinical biological markers in diagnosis of pleural effusions in children. Paediatr Respir Rev.

[40] Qiu L, Teeter LD, Liu Z, Ma X, Musser JM, Graviss EA (2006). Diagnostic associations between pleural and pulmonary tuberculosis. J Inf Secur.

[41] Kumar S, Agarwal R, Bal A, Sharma K, Singh N, Aggarwal AN, Verma I, Rana SV, Jha V (2015). Utility of adenosine deaminase (ADA), PCR & thoracoscopy in differentiating tuberculous & non-tuberculous pleural effusion complicating chronic kidney disease. Indian J Med Res.

[42] Ahluwalia G (2015). Exudative pleural effusion in chronic kidney disease: an aetiological dilemma. Indian J Med Res.

[43] Zhong XH, Ding J, Zhou JH, Yu ZH, Sun SZ, Bao Y, Mao JH, Yu L, Li ZH, Han ZM, Song HM, Jiang XY, Liu YL, Zhang BL, Xia ZK, Jin CH, Zhu GH, Wang M, Feng SP, Shen Y, Huang SM, Ma QS, Li HX, Wang XJ, Ichihara K, Yao C, Dong CY (2018). A multicenter study of reference intervals for 15 laboratory parameters in Chinese children. Zhonghua Er Ke Za Zhi.

[44] Bielsa S, Acosta C, Pardina M, Civit C, Porcel JM (2019). Tuberculous pleural effusion: clinical characteristics of 320 patients. Arch Bronconeumol.

