# Supplementary material for: Factors associated with negative pleural adenosine deaminase results in the diagnosis of childhood pleural tuberculosis
Source: BMC Infect Dis. 2021 May 25;21:473. doi: 10.1186/s12879-021-06209-1 (PMC8152150; doi:10.1186/s12879-021-06209-1)
Supplement: Supplementary file 1 — Additional file 1. [file 12879_2021_6209_MOESM1_ESM.docx]

**Factors associated with negative pleural adenosine deaminase results in the diagnosis of childhood pleural tuberculosis**

**Running title:** Factors associated with negative pleural ADA results

Xing-Fen Han^1^, Chao Han^2^, Feng Jin^3^, Jun-Li Wang^4^, Mao-Shui Wang^5^

Xing-Fen Han, 545073860@qq.com

Chao Han, 443167097@qq.com

Feng Jin, 2547875348@qq.com

Jun-Li Wang, [13907768146@163.com](mailto:13907768146@163.com)

Mao-Shui Wang, wangmaoshui@gmail.com

^1^ Department of Tuberculosis, Shandong Provincial Chest Hospital, Cheeloo College of Medicine, Shandong University, Jinan, Shandong, China;

^2^ Department of Geriatrics, Shandong Mental Health Center, Jinan, China;

^3^ Department of Thoracic Surgery, Shandong Provincial Chest Hospital, Cheeloo College of Medicine, Shandong University, Jinan, China;

^4^ Department of Lab Medicine, The Affiliated Hospital of Youjiang Medical University for Nationalities, Baise, China.

^5^ Department of Lab Medicine, Shandong Provincial Chest Hospital, Cheeloo College of Medicine, Shandong University, Jinan, Shandong, China;

**Address correspondence to:**

Jun-Li Wang, Department of Lab Medicine, Affiliated Hospital of Youjiang Medical University for Nationalities, Baise, China. E-mail: [13907768146@163.com](mailto:13907768146@163.com) (JLW).

Mao-Shui Wang, Department of Lab Medicine, Shandong Provincial Chest Hospital, 46# Lishan Road, Jinan city, PR China. 250013. TEL: +86 531-86568107; Fax: +86 531-86956760. E-mail: [wangmaoshui@gmail.com](mailto:wangmaoshui@gmail.com) (Mao-Shui Wang).

| Supplementary Table 1. Univariate analysis of the demographic and clinical data associated with negative pleural ADA results in childhood pleural TB. | | | | | | |
| --- | --- | --- | --- | --- | --- | --- |
|  | | Total (n) | Pleural ADA (≤40 U/L) | Pleural ADA (＞40 U/L) | P value | OR (95% CI) |
| N | | 84 | 17 (20.2%) | 67 (79.8%) |  |  |
| TB assays (pleural effusion) | |  |  |  |  |  |
|  | Culture | 49 (58.3%) | 9 (52.9%) | 40 (81.6%) | 0.595 |  |
|  | AFB smear | 1 | 0 | 1 | 1.000 |  |
|  | PCR | 22 (26.2%) | 6 (35.3%) | 16 (23.9%) | 0.444 |  |
| Vital Signs | |  |  |  |  |  |
|  | Temperature (℃) | 37.4±1.0 | 37.2±0.9 | 37.4±1.0 | 0.289 |  |
|  | Heart rate | 98.4±14.4 | 91.8±13.3 | 99.8±14.4 | 0.039 |  |
|  | Respiratory rate | 22.5±2.7 | 22.4±3.2 | 22.5±2.7 | 0.824 |  |
|  | Systolic pressure | 110.3±12.6 | 111.2±9.3 | 110.2±13.4 | 0.749 |  |
|  | Diastolic pressure | 68.5±8.8 | 68.3±8.9 | 68.7±8.9 | 0.923 |  |
| Medical history | |  |  |  |  |  |
|  | Treatment delay (days) | 57.1±168.0 | 127.2±341.6 | 39.0±74.4 | 0.185 |  |
|  | Contact history of TB | 10 (11.9%) | 3 (17.6%) | 7 (10.4%) | 0.418 |  |
|  | Transferred times | 2.1±0.9 | 2.3±1.2 | 2.0±0.9 | 0.348 |  |
|  | Transferred from a teaching hospital | 49 (58.3%) | 10 (58.8%) | 39 (58.2%) | 0.963 |  |
|  | Times of hospitalization | 1.8±1.3 | 1.7±1.2 | 1.8±1.4 | 0.782 |  |
|  | Surgical treatment | 11 (13.1%) | 3 (17.6%) | 8 (11.9%) | 0.536 |  |
| Comorbidity | |  |  |  |  |  |
|  | Pulmonary TB | 48 (57.1%) | 7 (41.2%) | 41 (61.2%) | 0.142 |  |
|  | Bronchial tuberculosis | 1 (1.2%) | 0 (0.0%) | 1 (1.5%) | 1.000 |  |
|  | Tuberculous lymphadenitis | 6 (7.1%) | 2 (11.8%) | 4 (6.0%) | 0.416 |  |
|  | Tuberculous meningitis | 1 (1.2%) | 0 (0.0%) | 1 (1.5%) | 1.000 |  |
|  | Milliary TB | 2 (2.4%) | 0 (0.0%) | 2 (3.0%) | 0.999 |  |
| Pleural cytology | |  |  |  |  |  |
|  | Pleural density (g/cm³) | 1.0183±0.0021 | 1.0186±0.0024 | 1.0183±0.0021 | 0.763 |  |
|  | White blood cell count (10^6^/L) | 3.6±3.1 | 5.4±5.7 | 3.4±2.6 | 0.372 |  |
|  | Mononuclear cell (%) | 81.9±22.5 | 73.0±24.7 | 82.2±23.1 | 0.854 |  |
|  | Polynuclear cell (%) | 18.1±22.5 | 27.0±24.7 | 17.8±23.0 | 0.852 |  |
| Blood analysis | |  |  |  |  |  |
|  | White blood cell (10^9^/L) | 7.4±2.7 | 7.3±2.3 | 7.4±2.8 | 0.922 |  |
|  | Red blood cell (10^12^/L) | 4.5±0.5 | 4.5±0.5 | 4.4±0.5 | 0.506 |  |
|  | Hemoglobin (g/L) | 120.8±11.3 | 121.5±11.9 | 120.7±11.2 | 0.780 |  |
|  | Hematocrit | 36.3±11.3 | 36.2±3.1 | 36.3±3.4 | 0.897 |  |
|  | Mean corpuscular volume (fL) | 82.7±4.6 | 81.3±4.7 | 83.1±4.5 | 0.161 |  |
|  | Mean corpuscular haemoglobin (pg) | 27.6±1.9 | 27.3±2.0 | 27.7±1.9 | 0.529 |  |
|  | Mean corpuscular haemoglobin concentration (g/L) | 333.3±12.3 | 335.6±9.8 | 333.8±12.8 | 0.406 |  |
|  | Platelet (10^9^/L) | 363.1±118.4 | 354.1±133.5 | 370.2±121.0 | 0.723 |  |
|  | Neutrophil (10^9^/L) | 5.5±8.0 | 4.7±1.9 | 5.7±8.9 | 0.671 |  |
|  | Lymphocyte (10^9^/L) | 1.7±0.9 | 1.8±0.7 | 1.7±1.0 | 0.747 |  |
|  | Monocyte (10^9^/L) | 0.8±0.4 | 0.7±0.3 | 0.8±0.4 | 0.477 |  |
|  | Coefficient of variation of red cell distribution width (%) | 13.4±1.2 | 13.5±1.5 | 13.4±1.2 | 0.611 |  |
|  | Erythrocyte sedimentation rate (mm/h) | 46.9±25.4 | 41.8±23.8 | 48.4±25.6 | 0.353 |  |
| Flow cytometry | |  |  |  |  |  |
|  | CD19+ (%) | 20.1±15.4 | 15.1±7.4 | 21.6±16.7 | 0.302 |  |
|  | CD3+ (%) | 64.4±12.3 | 66.3±10.7 | 63.8±12.6 | 0.597 |  |
|  | CD3+CD4+ (%) | 35.1±9.3 | 35.1±6.8 | 34.9±9.9 | 0.988 |  |
|  | CD3+CD8+ (%) | 25.4±9.7 | 27.5±8.0 | 24.8±10.0 | 0.449 |  |
|  | CD3-CD16+CD56+ (%) | 11.9±6.7 | 13.8±9.3 | 11.5±5.8 | 0.347 |  |
|  | CD4+/CD8+ (%) | 2.1±3.4 | 1.4±0.4 | 2.3±3.8 | 0.404 |  |
| ADA, adenosine deaminase; TB, tuberculosis; OR, odds ratio; CI, confidence interval; AFB, acid-fast bacilli; PCR, polymerase chain reaction. | | | | | | |
